# Rewiring Mitochondrial Metabolism for CD8^+^ T Cell Memory Formation and Effective Cancer Immunotherapy

**DOI:** 10.3389/fimmu.2020.01834

**Published:** 2020-08-27

**Authors:** Wenhui Li, Lianjun Zhang

**Affiliations:** ^1^Suzhou Institute of Systems Medicine, Suzhou, China; ^2^Center for Systems Medicine, Institute of Basic Medical Sciences, Chinese Academy of Medical Sciences and Peking Union Medical College, Beijing, China

**Keywords:** CD8 T cell, mitochondria, memory, cancer, immunotherapy

## Abstract

Memory T cells persist for long term to mediate robust recall response upon rechallenging with previous encountered pathogens. The memory T cell pool is highly heterogeneous based on distinct phenotypic, functional, and locational properties, and contains discrete subsets, which contribute to diverse immune responses. In this mini-review, we will briefly discuss the distinct subsets of memory T cells and then focus on mitochondria-related metabolic and epigenetic regulations of CD8^+^ T cell memory formation. In particular, we discuss many aspects of mitochondrial quality control systems (biogenesis, dynamics, etc.) in regulating CD8^+^ T cell fate decision and antitumor immunity. Importantly, targeting mitochondrial metabolism to boost T cell memory formation and metabolic fitness might represent an attractive strategy to improve cancer immunotherapy including CAR-T therapy.

## Memory T Cell Subsets

Memory T cells possess the property of long-term remembrance of priming antigens or pathogens, and evoke a rapid recall response with enhanced magnitude upon antigen reencounter (1). For many years, it is recognized that memory T cell pool is heterogeneous in terms of phenotypic markers, functional traits, epigenetic modifications, and metabolic features (2–4). In particular, human memory T cells were primarily categorized into central memory (Tcm) and effector memory (Tem) subsets, characterized by CD62^hi^CCR7^hi^ and CD62^low^CCR7^low^ phenotype, respectively, and with distinct functional/localization properties (5). Tcm cells have vast proliferative potential and reside in secondary lymphoid organs to invoke robust recall responses, which further differentiate toward effector memory or terminally differentiated effector progenies to protect against infections or undergo self-renew. Tem is commonly found in nonlymphoid tissues and circulate through blood continuously (6). Although Tem cells are less proliferative and could not persist for a long term, they are ready to provide immediate protection at infection sites via producing multiple cytotoxic molecules, including granzyme B (Gzmb), perforin, interferon gamma (IFNγ), tumor necrosis factor (TNF), etc. (5).

Recently, a subset of memory T cells called stem-cell-like memory T cells (Tscm) has been identified both in human and mice (7, 8). Tscm cells exhibit stem-cell-like properties including self-renewal and could further differentiate into memory or effector T cells, which is further validated by a single-cell adoptive transfer that gives rise to diverse progenies upon recall in mice (7–9). On the other hand, tissue-resident memory T cells (Trm) represent the important subset that is permanently embedded into tissues such as skin, lung, gut, brain, and even the tumor sites. Trm cells are characterized by CD103^hi^CD69^hi^CD62^low^CD27^low^ phenotype and act as the key player for local immune surveillance at the barrier tissues against reinfected pathogens and display accelerated immediate defense via rapid production of Gzmb, among many other effector molecules (10–13).

## Metabolic Regulation of Memory CD8^+^ T Cell Formation

The origin, formation, and maintenance of distinct subsets of memory T cells are tightly regulated by multiple extrinsic and intrinsic factors. In response to various stimuli, including virus, bacteria, parasites, fungi, and even tumor-derived mutated antigens, naive T cells undergo activation, expansion, and differentiation into distinct progeny of effector/memory T cells to eliminate infections (14). The molecular mechanisms underlying CD8^+^ T cell memory and effector differentiation have been illustrated at multiple levels including transcriptional regulation, epigenetic modification, and metabolic reprograming (15, 16). In this section, we will particularly focus on mitochondria-dependent metabolic reprograming in CD8^+^ T cell memory formation.

**Figure 1:**
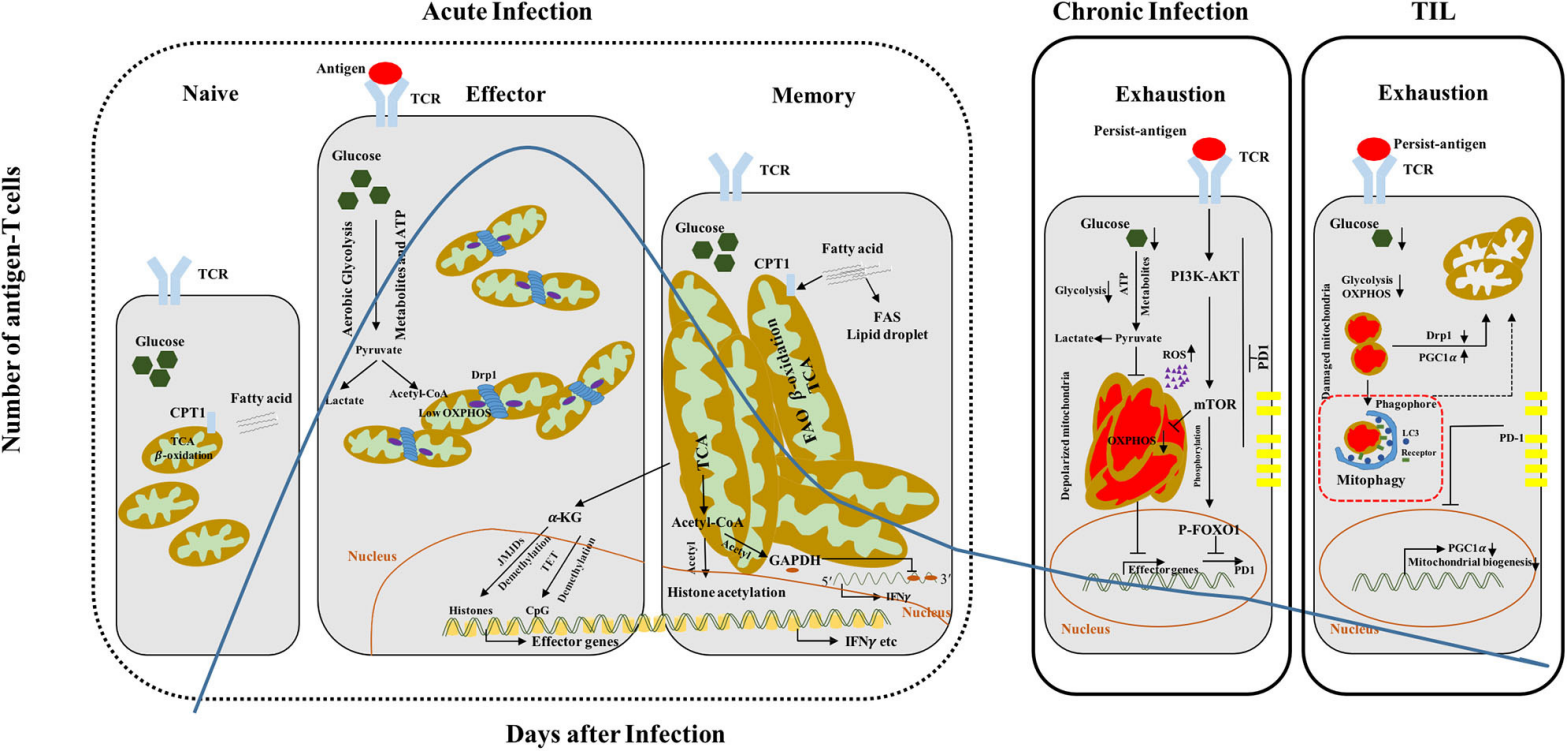
CD8^+^ T cell differentiation and functionality couples with dynamic metabolic programming. Quiescent naive T cells display small and fragmented mitochondria, which utilize OXPHOS and FAO (CPT1, the rate-limiting enzyme) to maintain their survival. Although effector T cells switch from OXPHOS to aerobic glycolysis to support clonal expansion and effector functions, they also show transiently increased mitochondrial mass accompanied with Drp1-mediated mitochondrial fission. In contrast, memory T cells harbor more fused and elongated mitochondria, which have high spare respiratory capacity (SRC), and predominantly utilize fatty acid to produce energy via β-oxidation, accompanied with fatty acid synthesis (FAS). The effector/memory differentiation process can also be regulated by epigenetic modifications including mitochondrial metabolites (acetyl-CoA and α-KG). Acetyl-CoA provide donor acetyl to histone and glyceraldehyde 3-phosphate dehydrogenase (GAPDH), which can be hyperacetylated respectively and promote interferon gamma (IFNγ) expression. α-KG acts as a cofactor of Jumonji C-domain-containing histone demethylases (JMJDs)/ten–eleven translocation (TET), and demethylates histone and DNA with the result of effector genes expression. Exhausted CD8^+^ T cells show multiple functional or structural alterations in mitochondria. Within CD8^+^ T cells during chronic infection, larger, structurally defective and depolarized mitochondria are accumulated and accompanied with high ROS production, leading to defective OXPHOS and loss of effector functions. TILs exhibit decreased mitochondrial mass but increased fragmented mitochondria with dysregulated structure and accumulation of ROS, resulting in defective glycolysis and OXPHOS. PD1 can negatively regulate the PGC1α and then inhibit mitochondrial biogenesis. Overexpression of PGC1α or knockdown Drp1 can recover the mitochondrial defects and functions and promote T cell effector functions and antitumor capacity. TCR, T cell receptor; CPT1, carnitine palmitoyltransferase 1; TCA, tricarboxylic acid cycle; acetyl-CoA, acetyl coenzyme A; FAO, fatty acid oxidation; FAS, fatty acid synthesis; OXPHOS, oxidative phosphorylation; PGC1α, peroxisome-proliferator-activated receptor gamma coactivator 1α.

Naive T cells remain in quiescent state and preserve their survival mainly via oxidative phosphorylation (OXPHOS). Upon TCR activation, naive CD8^+^ T cells first undergo extensive size increase, followed by proliferative burst and acquisition of cytotoxic functions, which are accompanied by reprogramming of anabolism and catabolism (17, 18). In particular, the prominent aerobic glycolysis takes over within effector CD8^+^ T cells with relatively low rate of OXPHOS (19) (Figure 1). To this end, a large number of intermediate metabolites produced via glycolysis are engaged to build the macromolecules and support the proliferation burst of effector T cells. Interestingly, memory CD8^+^ T cells rely heavily on OXPHOS to support their survival and function, in which the fatty acid is predominantly used to fuel fatty acid β-oxidation (FAO) and maintains spare respiratory capacity (SRC). Surprisingly, memory CD8^+^ T cells prefer to utilize a “futile cycle” of *de novo* fatty acid synthesis (FAS) and lysosome-based lipid storage in order to maintain long-term survival and supply adequate ATP immediately during antigen rechallenge, rather than direct uptake of fatty acids from the environment (20) (Figure 1). Although both naive and memory T cells rely on OXPHOS, naive T cells harbor less mitochondrial mass and lower SRC as compared to memory T cells (21, 22). Furthermore, CD8^+^ Trm cells generated from viral-infected skin exhibit increased lipid metabolism dependent on fatty-acid-binding proteins 4 and 5 (FABP4 and FABP5) mediated exogenous lipid uptake and transport in both mouse and human tissues (23).

The mammalian target of rapamycin (mTOR) and AMP-activated protein kinase (AMPK) signaling pathway, the key sensors of intracellular energy status, are also critical regulators of memory CD8^+^ T cell formation. mTOR complex I (mTORC1) activation is required for protein synthesis and generation of the biomolecules for proliferation. Interestingly, inhibition of mTORC1 by rapamycin promotes FAO and memory CD8^+^ T cell formation, which was also confirmed by the findings that AMPK (the upstream kinase of mTOR signaling) activator metformin increases the pool of memory CD8^+^ population via boosting OXPHOS rate (24–26). Of note, complete ablation of mTORC1 activity via genetic deletion of Raptor impairs both effector and memory differentiation. Interestingly, we and others recently demonstrated that functional deficiency of mTORC2 leads to enhanced CD8^+^ T cell memory formation, which was associated with increased mitochondrial metabolism and FAO (26, 27). Similarly, we found that fine tuning of the mTOR signaling rather than Wnt signaling activation is responsible for the generation of human Tscm (28). Moreover, it has been shown that restrained glycolytic activity or enhanced FAO, which are achieved by inhibition of AKT or overexpression of rate-limiting β-oxidation enzyme CPT1α, favors the formation of memory CD8^+^ T cells and restricts the effector differentiation (22, 29). Consistently, enhanced glycolytic flux by overexpressing phosphoglycerate mutase-1 (*Pgam1*) in CD8^+^ T cells blocks its memory formation (30). Conversely, inhibition of glucose metabolism in the presence of Hk2 inhibitor 2-deoxy-d-glucose favors memory CD8^+^ T cells' generation and augments antitumor immunity (30). Altogether, those findings strongly suggest that genetic or pharmacological modulation of T cell metabolism may dictate T cell fate decision and reprogram the effector/memory lineage specification.

## Mitochondrial Regulation of CD8^+^ T Cell Memory Formation or Exhaustion

Mitochondria are dynamic organelles that undergo fission and fusion to maintain homeostasis and emerge as the hub of innate and adaptive immunity. For instance, mitochondrial DNA (mtDNA) contains plenty of CpG islands, which can be recognized by TLR9 and trigger nuclear factor kappa B (NF-κB) activation and proinflammatory response (31, 32). Mitochondrial antiviral signaling (MAVS), the mitochondrial out-membrane protein, can protect against RNA virus infection via interacting with virus RNA sensor RIG-I and promoting NF-κB and interferon regulatory factor (IRF) activation (33, 34). Herein, we discuss mainly the role of mitochondria in regulating memory CD8^+^ T cell formation and functionality.

CD8^+^ T cells with distinct activation or functional states harbor different mitochondrial morphologies to fulfill their metabolic demands and biological functions. Naive CD8^+^ T cells are characterized by small and fragmented mitochondria, accompanied with relatively lower level of basal energy consumption through mitochondrial OXPHOS (35). Effector T cells have more punctate mitochondria as indicated by increased mitochondrial fission, which lead to lower OXPHOS and higher aerobic glycolytic rate (36) (Figure 1). Moreover, mitochondrial fragmentation also promotes ROS production, which is required for acquisition of effector functions in activated T cells (37). On the other hand, memory CD8^+^ T cells exhibit increased mitochondrial fusion and mass, which display larger network of elongated mitochondria and may maintain SRC for efficient utilization of fatty acid and rapid proliferation upon recall (36) (Figure 1). Mdivi-1, an inhibitor of mitochondrial fission, enhances respiratory capacity and promotes the formation of memory T cell pool. Consistently, knockdown of Drp1 or overexpression of OPA1, the key mediators of mitochondrial fission and fusion, respectively, promotes CD8^+^ T cell memory generation (36, 38).

In addition to mitochondrial dynamics, mitochondrial quality can also be regulated by mitochondrial biogenesis, mitophagy, and mitochondrial unfolded protein response (UPR^mt^) (39). T cells undergo exhaustion during chronic viral infection or within the tumor microenvironment (TME), which was characterized by gradual loss of proliferative capacity and effector function, and accompanied by accumulation of dysfunctional mitochondria (40, 41). Of note, exhausted CD8^+^ T cells generated upon chronic lymphocytic choriomeningitis virus infection exhibit higher rate of depolarized mitochondria, larger size, and increased ROS level, whereas tumor-infiltrating lymphocytes (TILs), also characterized by exhausted phenotype, show decreased total mitochondrial mass and increased depolarized mitochondria, with fragmented morphology and low level of ROS (42, 43) (Figure 1). Furthermore, dysregulation of mitochondrial homeostasis, indicated by increased ROS level and lower mitochondrial potential, has been observed in exhausted CD8^+^ T cell from patients with chronic hepatitis B virus (HBV) infections. Scavenger ROS in exhausted T cells by mitochondrion-targeted antioxidants and recovered mitochondrial metabolic capacity by interleukin-12 are confirmed effective approaches to boost antiviral CD8^+^ T cell functions (44, 45). In contrast to B16 melanoma infiltrated lymphocytes, human CD8^+^ TILs from clear cell renal cell carcinoma (ccRCC) patients display small, fragmented, and hyperpolarized mitochondria companied with high level of ROS and increased mitochondrial mass with Mitotracker Green (MTG) staining (46). Consequently, the mitochondria demonstrate distinct phenomena in CD8^+^ TILs from different types of tumor, which may be attributed to tumor heterogeneity and complicated microenvironment. Although TILs showed distinct mitochondrial morphologies, mitochondrial dysfunction and metabolic deficiencies are common features observed in most TILs. Furthermore, a large number of total mitochondria and depolarized mitochondria were observed in early exhausted CD8^+^ T cells during chronic LCMV clone 13 infection, which can be reversed by PGC1α (the master regulator of mitochondrial biogenesis) overexpression (42). In contrast, transduction of PGC1α into CD8^+^ TILs promotes mitochondrial biogenesis to maintain the mitochondrial quality and increase the mass. Among these distinct observations, the quality and quantity of mitochondria are both critical parameters in response to diverse stimuli. It is possible that the turnover of the accumulated damaged mitochondria in early exhausted T cells could be stimulated by PGC1α overexpression, which acts to decrease the depolarized mitochondria and in turn maintain the quality and integrity of mitochondria. Intriguingly, it was shown that PD1 could directly suppress PGC1α expression (43). Within the TME, PGC1α expression was repressed in TILs due to activation of AKT signaling and blockade of Foxo1, which is also confirmed by chronic LCMV-infection model. Therefore, neutralization of PD1 boosts PGC1α expression, and overexpression of PGC1α can improve healthy mitochondrial mass and function, and recover antitumor abilities of exhausted T cells (43).

Autophagy is important for maintaining cell homeostasis via highly selective self-degradation process to clear infected pathogens, aggregated proteins, and damaged or superfluous organelles (mitochondria, ribosome, peroxisome, etc.) (47). Interestingly, T-cell-specific deficiency of Atg5 or Atg7 impairs memory formation without damaging effector differentiation. Along the same line, T cells with Atg7 deletion harbor increased mitochondrial mass and enhanced ROS level, due to failure of removal of the damaged mitochondria (48). These findings highlight the significance of mitochondrial homeostasis through autophagy during CD8^+^ T cell memory differentiation and maintenance. Mitophagy, an important regulatory mechanism to maintain the mitochondrial quality and integrity, can selectively eliminate dysfunctional or superfluous mitochondria (49). Nevertheless, the role of mitophagy is less studied and remains largely unknown during CD8^+^ T cell memory formation. The mitophagy receptor BNIP3L (also called NIX) can directly interact with LC3 and recruit autophagic vacuole to mitochondria, followed by fusion with lysosome and degradation of dysfunctional mitochondria (50). A recent study demonstrated that NIX-mediated mitophagy could promote CD8^+^ T cell effector memory differentiation by preventing HIF1α accumulation and maintaining long-chain fatty acid metabolism (51). Given the accumulation of small fragmented mitochondria in CD8^+^ T cells within the TME, it remains to be investigated how does mitophagy regulate T cell exhaustion and antitumor immunity.

## Epigenetic Basis of the Memory T Cell Recall Response

Multiple pairs of transcription factors have been well demonstrated to regulate CD8^+^ T cell effector or memory differentiation (3). For instance, the transcriptional factor T-bet is required for the generation of KLRG^hi^ terminated effector T cells, whereas Eomes foster memory T cell generation (52, 53). Moreover, Id2/Id3 and Blimp-1/Bcl-6 are also critical regulators of CD8^+^ T cell effector or memory progenies (54, 55). Besides the transcriptional regulation, epigenetic modifications including chromatin remodeling, DNA methylation, and histone modifications (methylation, acetylation, phosphorylation, ubiquitination, etc.) represent another important layer to modulate gene expression patterns and dictate CD8^+^ T cell fate decision (15). DNA methylation predominantly occurs at clusters of CpG dinucleotides, which is also called CpG island and located in gene promoters (56). High frequency of CpG methylation acts as a repressive gene expression marker and affects the chromatin accessibility and transcription factor docking. In memory CD8^+^ T cells, DNA methylation at the promoter of *IFN*γ, *Gzmb*, and *IL2* loci is increased and correlates with suppressed gene expression (57). However, *Tcf7*, a critical transcription factor in memory and naive T cells, and other memory genes (*CD62L* and *CD127*), are demethylated and show enhanced expression as compared to activated or effector cells (58). On the other hand, effector T cells show dramatically increased expression of multiple effector genes such as *IFN*γ, *TNF, IL2*, and *Gzmb*, due to reduced level of methylation at their promoter or enhancer region (59). Although the methylation landscape of memory T cells is similar with that of naive T cells, memory T cells are capable of maintaining their epigenetic landscape at effector genes loci, but fewer methylations occur at the same genes in naive T cells, indicating that memory T cells retain an effector-like signature in order to rapidly acquire cytotoxic ability and formation of effector T cells upon recall (60, 61).

Histone modification represents another important layer of chromatin structure modulation, which impacts subsequent transcription factor docking and gene expression. Hyperacetylation, such as H3K9Ac, may improve the chromatin accessibility and enhance the effector gene expression in memory T cells upon recall. Genome-wide analysis of histone methylation shows diverse methylated sites at arginine and lysine; the dimethylated or trimethylated H3K4, H3K9, and H3K27 on the gene loci of effector/memory T cells are involved in the formation and maintenance of T cell memory. Generally, the expression level of effector genes is associated with higher level of H3K4me3 and low level of H3K27me3 in memory T cells upon recall. Thus, these observations support the notion that memory T cells are epigenetically imprinted to mediate an accelerated recall response.

Mitochondrial intermediate metabolites such as citrate, acetyl-CoA, and α-ketoglutarate (α-KG) are derived from Krebs cycle and involved in epigenetic modifications including histone acetylation. Accumulated acetyl-CoA can provide donor acetyl for subsequent histone acetylation, and IFNγ will be transcribed abundantly due to hyperacetylation of its promoter in CD4^+^ T cells (62). Moreover, acetyl-CoA is also used to acetylate glyceraldehyde 3-phosphate dehydrogenase (GAPDH), which inhibits IFNγ translation via binding the 3′ unfolded protein response (UPR) of its messenger RNA (mRNA), promotes IFNγ production by blocking the interaction (63). During acute bacterial infection, acetate is accumulated in the serum and subsequently uptake by memory CD8^+^ T cells to accelerate acetyl-CoA production. High concentration of acetyl-CoA provides acetyl group to GAPDH and boost its enzyme activity, which enhances glycolytic flux and cytotoxic capacity (for instance, IFNγ production) (64) (Figure 1). Furthermore, acetate promotes IFNγ production via acetyl-CoA synthesis in glucose-restricted CD8^+^ T cells and tumor-exposed exhausted T cells, which is attributed to histone acetylation and transcriptional accessibility. Acetyl-CoA synthetase 2 (ACSS2), which can convert acetate to acetyl-CoA, also enhances CD8^+^ T cell effector functions and IFNγ expression *in vivo* (65). In addition, α-KG can act as a cofactor for Jumonji C-domain-containing histone demethylases (JMJDs) and ten–eleven translocation (TET) 5-methycytosine hydroxylases, which mediate histone and DNA demethylation, respectively. Levels of α-KG or α-KG–succinate ratios can therefore remodel the epigenomes and gene expression in CD4^+^ or CD8^+^ T cells, and accumulation of α-KG drives gene expression associated with effector function and cell differentiation (66, 67). Isocitrate dehydrogenase 1 (IDH1) mutation was also determined to produce α-hydroxyglutarate instead of α-KG, and this results in a block of cell differentiation due to inhibition of histone demethylation (68). Moreover, S-2-hydroxyglutarate, the metabolite enantiomer of 2-hydroxyglutarate, occurs in IDH1/2 mutations or damaged mitochondria and hypoxia treatment, increases the CD8^+^ T cell recall capacity, and promotes the persistence of adoptively transferred CD8^+^ T cells and antitumor immunity via modulating DNA/histone methylation and HIF1α stability (69).

## Rewiring Mitochondrial Metabolism: A Novel Approach for Immunotherapy of Cancer

Unlike traditional surgical resection, radiation, chemotherapy, and targeted therapies, immunotherapeutic strategies provide novel opportunities for long-term protection against cancer development and recurrence. Immune checkpoint blockade (ICB) and chimeric antigen receptor T cell (CAR-T) therapy represent two successful strategies to treat patients across multiple types of cancer in clinics (70, 71). Given their enhanced expression in TILs, antibodies against either CTLA4, PD1, or in combination can be used to partially recover the impaired T cell functionality and lead to drastic tumor regression both in mouse models and human cancer patients, but only a small fraction of patients benefit from the treatment (72). Although the CAR-T therapy achieved great success in certain hematological cancers, it remains challenging to treat the majority of malignant solid tumors with current CAR-T technology; one key reason is that infiltrated CAR-T undergo metabolic or functional exhaustion in the TME (73). Thus, there is an urgent need to develop novel strategies to enhance CD8^+^ T cell functionality within the TME. Here, we discuss how can we improve the efficacy of antitumor immunity by modulating mitochondrial metabolism within T cells. In particular, we present the potential mitochondrial targeting strategies that could boost T cell immunity against cancer.

Within the TME, effector T cells are under severe metabolic stress such as nutrient deprivation (low glucose, lower amino acids, etc.), hypoxia, and accumulation of lactate acid and ROS (74, 75). These disadvantages result in dramatic metabolic changes in TILs and may facilitate the evasion of the tumor cells from immune surveillance. To overcome the accumulation of small fragmented mitochondria in CD8^+^ T cells within the TME, increased mitochondria biogenesis via PGC1α overexpression in T cells boosts the mitochondrial mass and metabolic capacity (glutamine metabolism and OXPHOS) and enhances antitumor immunity (43, 76). Moreover, PGC1α overexpression-mediated antitumor immunity may be combined with anti-PD1 antibody, as suggested by significantly regressed tumor volume with combinatory treatment (76). Bezafibrate, a PGC1α agonist, has been shown to boost antitumor immunity via upregulating mitochondrial OXPHOS and inhibition of apoptosis in MC38-bearing mouse tumor model treated with PD1 blockade (77). Thus, pharmacological induction of mitochondrial biogenesis in T cells may represent a potential therapeutic target for cancer immunotherapy. Of note, it requires further investigation regarding the role of mitophagy in regulating T cell exhaustion and antitumor immunity, in particular to understand the crosstalk between mitophagy and mitochondrial biogenesis in modulating T cell metabolic fitness and functionality. Furthermore, costimulatory or coinhibitory molecules also critically regulate T cell metabolism and mitochondrial phenotype (78). For instance, CD28 signaling during T cell activation stimulates aerobic glycolysis and promotes mitochondrial fusion (79). 4-1BB costimulation can also augment glycolysis via promoting the expression of glucose transporters to support CD8^+^ T cell proliferation (80). Moreover, 4-1BB costimulation enhanced mitochondrial fusion and biogenesis, which are independent of PGC1α-mediated pathways and p38-MAPK signaling, and resulted in improving metabolic sufficiency and antitumor immunity in CD8^+^ TILs (81). Furthermore, overexpression of 4-1BB in TILs promotes mitochondrial respiration capacity, associated with increased OPA-1-induced mitochondrial fusion and PGC1α-mediated mitochondrial biogenesis. Indeed, those modified T cells better survive and function under metabolic stress at the TME and trigger enhanced antitumor immunity (81, 82). On the other hand, PD1 enhances FAO via upregulating Cpt1α, and CTLA4 restricts glucose metabolism by reducing AKT phosphorylation in CD4^+^ T cells (83, 84).

In addition, the glycolytic capacity and cytokine production of TILs are dampened given the competition for nutrient consumption between tumor cells and effector T cells within the TME (74). Lactate, the product of glycolytic pathway and mostly derived from tumors, directly suppresses T cell proliferation and cytotoxic activity (85). Thus, tumor-imposed metabolic restrictions attenuate the effector functions of TILs and antitumor immunity. TILs suffer from low glucose in the TME and also display fragmented mitochondria, and administration of Mdivi-1 to inhibit mitochondrial fission or knockdown of Drp1 leads to fused mitochondria and increased metabolic fitness in TILs, which thus display superior antitumor effects (36). Thus, reprogramming mitochondrial dynamics by inhibiting mitochondrial fission or promoting fusion may increase the OXPHOS and T cell immunity and represent a potential target for rescuing the effector functions of exhausted CD8^+^ T cells.

Adoptive cell transfer (ACT) of tumor-antigen-specific T cells could mediate considerable antitumor effects in clinics. Zelig Eshhar's lab first reported CAR-T technology consisting of a single-chain antibody for recognizing the targeted antigen and signaling transduction domain for T cell activation and acquisition of effector functions. To this end, this genetically modified T lymphocytes could directly target and kill the tumor cells that express the cognate antigen of designed antibody (86). Later, the costimulatory CD28 or 4-1BB functional domains were included in the CAR-T engineering and enhanced therapeutic efficacy (87). Thus, the addition of costimulatory domain to CAR is essential for boosting CAR-T activities. Although ICB is an effective approach for treating certain cancers, TILs also exhibit metabolic insufficiency and mitochondrial dysfunction (decreased mitochondrial mass, fragmentation, lower OXPHOS, etc.) (43). In this regard, development of strategies that could recover or boost mitochondrial functions is important to promote T cell or CAR-T antitumor immunity. Consistently, human CAR-T armed with 4-1BB domain show higher rate of expansion, survival, and superior antitumor immunity *in vivo* due to enhanced mitochondrial mass and fitness. In particular, 4-1BB domain can enhance mitochondrial biogenesis, fatty acid β-oxidation, and SRC (88). Interestingly, a recent study demonstrated that CAR-T with herpes virus entry mediator (HVEM)-derived costimulatory domain exhibits higher mitochondrial respiration and glycolysis compared with CD28 and 4-1BB, which accounts for increased cytotoxic capacity and alleviated exhausted signature (89). Taken together, constructing new generation CAR-T armed with the optimal costimulatory domain to improve mitochondrial quality will be the key to mediate effective antitumor immunity.

Altogether, we believe that mitochondria may act as a key hub in determining CD8 T cell memory differentiation, maintenance, or functional decline (exhaustion) within the TME. Modulating mitochondrial biogenesis, dynamics (fusion/fission), and clearance of dysregulated mitochondria via mitophagy is highly relevant for designing immunotherapeutic strategies against cancer. In addition, rewiring the metabolic communication between distinct cell types within the TME may largely improve the successful rate of CAR-T therapy in solid cancers. Given that certain mitochondrial metabolites are important regulators of epigenetic modifications, fine-tuning mitochondrial quantity/quality/integrity is required for achieving better metabolic fitness or boosting T cell antitumor immunity via epigenetic remodeling the T cell exhaustion signature.

## Author Contributions

WL and LZ: conception, writing and revision of the manuscript. All authors listed have made a substantial, direct and intellectual contribution to the work, and approved it for publication.

## Conflict of Interest

The authors declare that the research was conducted in the absence of any commercial or financial relationships that could be construed as a potential conflict of interest.

## Abbreviations

Tcm: central memory T cell
Tem: effector memory T cell
Tscm: stem cell memory T cell
Trm: resident memory T cell
TCR: T cell receptor
OXPHOS: oxidative phosphorylation
UPR^mt^: mitochondrial unfolded protein response
ROS: reactive oxygen species
FAO: fatty acid oxidation
FAS: fatty acid synthesis
SRC: spare respiratory capacity
TILs: tumor-infiltrating lymphocytes
ICB: immune checkpoint blockade
ACT: adoptive cell transfer
CAR: chimeric antigen receptor
ECAR: extracellular acidification rate.
